# Analysis of the Relationship between the Levels of Androgens and Biochemical Bone Markers in Men Aged 60–75 Years

**DOI:** 10.3390/ijerph17010106

**Published:** 2019-12-22

**Authors:** Aleksandra Rył, Aleksandra Szylińska, Alina Jurewicz, Andrzej Bohatyrewicz, Tomasz Miazgowski, Iwona Rotter

**Affiliations:** 1 Department of Medical Rehabilitation and Clinical Physiotherapy, Pomeranian Medical University in Szczecin, Żolnierska 48, 71-210 Szczecin, Poland; aleksandra.ryl@pum.edu.pl (A.R.); iwrot@wp.pl (I.R.); 2 Department of Orthopedics, Traumatology and Orthopedic Oncology, Pomeranian Medical University in Szczecin, Unii Lubelskiej 1, 71-252 Szczecin, Poland; ala.jurewicz@wp.pl (A.J.); bohatyrewicz@orthopedics.pl (A.B.); 3 Department of Hypertension and Internal Medicine, Pomeranian Medical University in Szczecin, Unii Lubelskiej 1, 71-252 Szczecin, Poland; miazgowski@interia.pl

**Keywords:** biochemical bone markers, levels of androgens, aging men

## Abstract

*Introduction*: The purpose of this study was to analyze the relationship between the parameters of bone turnover and the levels of hormonal parameters, such as total testosterone (TT), bioavailable and free testosterone (FT), and estradiol (E2) in men. *Material and methods*: The study group included 63 men with testosterone deficiency syndrome (TDS). The control group consisted of 112 patients without TDS. Enzyme-linked immunosorbent assay (ELISA) was used to determine the levels of osteocalcin (OC), parathyroid hormone (PTH), E2, sex hormone binding globulin (SHBG), dehydroepiandrosterone sulphate (DHEAS), insulin (I), Serum CrossLaps (CtX-I), human procollagen I N-terminal peptide (PINP), and TT. Bone mineral density (BMD) was measured by dual-energy X-ray absorptiometry. *Results*: The groups with TSD and without TDS differed in terms of the following parameters: body weight (*p* = 0.001), BMI (*p* = 0.003), TT (*p* = 0.001), FT (*p* = 0.004), bioavailable testosterone (*p* = 0.001), E2 (*p* = 0.003), SHBG (*p* = 0.003), and PINP (*p* = 0.004). In the group without TDS, higher PINP levels were accompanied by higher levels of E2 (beta = 0.360, *p* = 0.002) and TT (beta = 0.389, *p* = 0.001). In the group without TDS, PINP was positively correlated with E2 (beta = 0.726, *p* <0.001). Patients with TDS had significantly lower PINP levels (*p* < 0.004). *Conclusions*: Analysis of sex hormones and biochemical bone markers in reflecting the quality of the bone tissue in men may suggest a relationship between these parameters. Nevertheless, further research based on a larger sample size is necessary to better describe this relationship.

## 1. Introduction

The relationship between the levels of androgens and bone mineral density (BMD) is still a subject of research. It has been demonstrated that androgen receptors―expressed in osteoblasts, osteoclasts, osteocytes, and pluripotential mesenchymal bone marrow stroma―influence bone function and metabolism [1]. One of the causes of bone mass decline in men is an age-related decrease in the level of testosterone (T) [1,2,3]. Testosterone deficiency syndrome (TDS) is a condition that not only contributes to lower libido and *erectile* dysfunction, but also negatively affects other aspects of men’s lives. 

A decrease in total testosterone (TT) levels results from the reduced activity of the hypothalamus-pituitary-gonad axis. T deficiency exacerbates a negative calcium balance, and decreases the levels of active vitamin D metabolites [4]. Both T and 5α-dihydrotestosterone have an effect on osteoblast longevity and differentiation [5]. They inhibit apoptosis of osteoblasts, stimulate production of interleukin-1β, and increase the level of the mitogenic fibroblast growth factor [6]. Androgens also suppress the activity of IL-6, which is a cytokine that activates the process of bone resorption [5]. 

What should also be taken into account is an indirect impact of TT on the bone tissue. TT in the bone tissue is aromatized (by P450 aromatase) to estrogen [7]. Estradiol (E2) has been proved to suppress osteocyte apoptosis by reducing the levels of cytokines (including IL-1 and IL-7) that activate bone resorption. It also inhibits the receptor activator of nuclear factor kappa-B ligand (RANKL). Furthermore, E2 increases synthesis of transforming growth factor alpha (TGF-α), which inhibits resorption of osteoclasts [5]. Studies show that men with aromatase deficiency commonly suffer from osteopenia and osteoporosis [8] and selective blockade of aromatase activity leads to a decrease in BMD [9,10].

In the article presented here, we analyzed the relationship between TDS and the levels of hormones in men, the parameters of bone turnover, and density and bone mineralization. Both androgen deficiency and the reduction of bone mass are related to patients’ age, therefore searching for markers and treatment methods common to both diseases may be of clinical significance. The purpose of this study was to analyze a relationship between the parameters of bone turnover and the levels of hormonal parameters, such as TT, bioavailable and free testosterone (FT), and E2 in healthy aging men.

## 2. Material and Methods

### 2.1. Study and Control Groups

The research material came from 175 subjects, aged between 60 and 75 years, hospitalized due to osteoarthritis. They were patients of the Clinic of Orthopedics, Traumatology and Orthopedic Oncology, Pomeranian Medical University in Szczecin, Poland. Patients were also recruited from the outpatient clinic. The exclusion criteria were: type 1 and 2 diabetes, cancer diseases, active alcohol use disorder, liver or renal insufficiency, *New York Heart Association* (*NYHA*) class *III* or *IV heart failure*, and taking agents potentially affecting bone metabolism, mineral supplementation, neuroleptics, chemotherapeutic agents, immunosuppressive drugs, steroids, or antidepressants. 

The research protocol was approved by the Bioethical Committee of the Pomeranian Medical University in Szczecin (approval no. KB-0012/155/16). 

### 2.2. Clinical Examination

To determine metabolic parameters, 9 mL blood samples were collected from participants on an empty stomach from the *ulnar vein*. Serum was kept in Eppendorf test tubes in a freezer at temperature of −20 °C (no longer than for three months). Weight and height measurements were taken in both groups. The levels of albumin were determined in the patients’ blood serum. 

The ELISA method (DRG Medtek, Warszawa, Poland) was used to determine in serum the levels of osteocalcin (OC), parathyroid hormone (PTH), E2, sex hormone binding globulin (SHBG), dehydroepiandrosterone sulphate (DHEAS), insulin (I), Serum CrossLaps (CtX-I), human procollagen I N-terminal peptide (PINP), and TT.

We decided to determine selected markers because OC is a noncollagen protein responsible for bone mineralization and is produced by osteoblasts, odontoblasts, and chondrocytes. OC also increases insulin secretion. PTH is a hormone that stimulates the proliferation of osteoclasts, CtX-I is a marker of bone resorption, and PINP is regarded as a marker of bone formation. We decided to determine testosterone levels because it is aromatized to estrogen and reduced to dihydrotestosterone (DHT) in bone tissue. Activation of androgen receptors (AR) is essential for normal development of the trabecular bone. Aromatization and estrogen receptors (ER) play a less significant but also important role. An increase in periosteal bone tissue mainly depends on the activation of AR, but it is optimal when both AR and ER are activated. Most of the circulating *testosterone in the body* is bound to SHBG. The levels of DHEAS were determined because a change in their level is supposed to play a role as a protective mechanism against osteoporosis. 

### 2.3. The Levels of Androgens Calculations

The level of FT was determined from TT, *SHBG, and albumin* levels using the formula developed by Vermeulen [11,12]. The level of bioavailable testosterone (bioT) *was calculated using the formula developed by* Morris at al. [12,13].

### 2.4. Criteria for a Diagnosis of Testosterone Deficiency Syndrome

TDS was diagnosed on the basis of the guidelines resulting from the consensus reached by the International Society of Andrology (ISA), the International Society for the Study of the Aging Male (ISSAM), the European Association of Urology (EAU), the European Academy of Andrology (EAA), and the American Society of Andrology (ASA) in the year 2000 [14]. The patients with TT levels below 2.5 ng/mL or in the range of 2.5–3.5 ng/mL, showing clinical symptoms assessed by Morley’s questionnaire, were assigned to the group with TDS. The study group included 63 men with TDS. The control group consisted of 112 patients without TDS. 

### 2.5. Bone Mass Measurements

BMD was measured by dual-energy X-ray absorptiometry (DXA; GE Lunar Prodigy Advance, Madison, WI, USA; software enCORE version 8.8) using an automatic scan mode. BMD was measured in the complete skeleton. The BMD values were expressed in grams per square centimeter. BMD spine analysis was performed in the lumbar segments 1–4 (L1–L4), and BMD hip analysis involved the whole femoral bone neck.

### 2.6. Statistical Analysis

Statistical analysis was performed using SPSS Statistics, v. 13.1. (StatSoft, Inc. Tulsa, OK, USA). Primary statistics, including mean, standard deviation, and ranges, were used for the group characteristics and normality of distribution was assessed by the Shapiro–Wilk test. Descriptive statistics included means ± standard deviation (SD) for continuous variables and frequency distributions for categorical variables. Variables with normal distribution were compared using the parametric Student’s *t*-test; otherwise, the non-parametric Mann–Whitney U test was used.

Multivariate linear regression analysis was performed and a *receiver operating characteristic* (ROC) curve was created. The significance level was set as *p* ≤ 0.05. 

## 3. Results

In our study, the mean t score in the group of patients with TDS was 0.42 and 1.18 in the group of patients without TDS. The difference was not statistically significant. The relationship between biochemical anthropometric parameters in the groups of patients with TSD and the group without TSD was analyzed (Table 1) and it was found that these groups differed in terms of the following parameters: body weight (*p* = 0.001), BMI (*p* = 0.003), TT (*p* = 0.001), FT (*p* = 0.004), bioT (*p* = 0.001), E2 (*p* = 0.003), SHBG (*p* = 0.001), and PINP (*p* = 0.004).

Next, multivariate linear regression analysis was performed (Table 2). The analysis demonstrated a relationship between PINP and hormonal parameters. In the group without TDS, higher PINP levels were accompanied by higher levels of E2 (beta = 0.360, *p* = 0.002) and TT (beta = 0.389, *p* = 0.001). In the group without TDS, PINP was positively correlated with E2 (beta = 0.726, *p* < 0.001 *). It is also worth emphasizing that the analysis of the relationship between PINP, FT, and BioT in the group of patients without TDS demonstrated values at the limit of statistical significance. 

**Figure 1:**
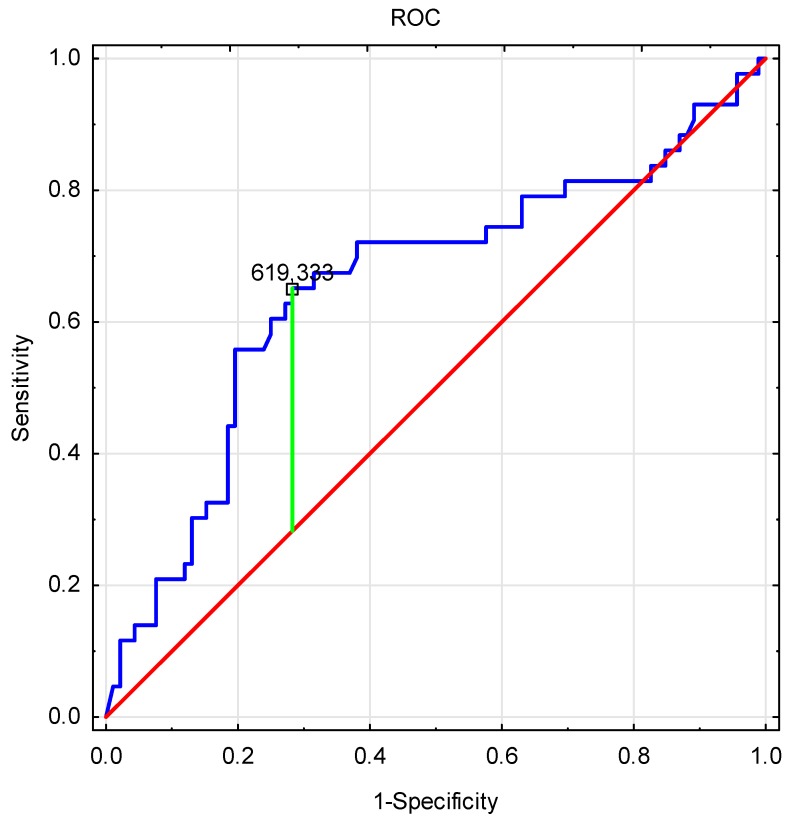
Receiver Operating Characteristic Curve (ROC) curve for PINP with regard to TDS.

Patients with TDS had significantly lower PINP levels (*p* < 0.004) (Table 1). Therefore, in further analysis we searched for a cut-off point for a continuous PINP parameter with regard to TDS. The Receiver Operating Characteristic Curve (ROC) for the PINP parameter with regard to TDS was analyzed (Figure 1). The cut-off point was 619.333 with an area under the curve (AUC) of 0.656 (0.550–0.762) and *p* = 0.004. The sensitivity and specificity of the point were 0.651 and 0.717, respectively. 

The groups with and without TDS were divided into subgroups according to the PINP cut-off point of 619.333 (Table 3). We compared these subgroups, taking into account values > 619.333. The subgroups differed in terms of the relationship between the levels of TT (*p* = 0.006) and BMD (*p* = 0.032). This relationship was only found in the group of patients without TDS. The relationship between the levels of E2 and BMD was observed in both subgroups (with TDS: *p* = 0.01; without TDS: *p* = 0.007).

## 4. Discussion

Studies of the association between the levels of androgens and the parameters of bone turnover metabolism in aging men have thus far provided ambiguous results [15]. It has been demonstrated that about 70% of men with osteoporosis have TDS, which, however, is only observed in involutional osteoporosis [16]. It is also worth emphasizing that both young and old men, after surgical or pharmacological castration, suffer from a rapid decline in mineral bone density (observed as quickly as after six to nine months), which is accompanied by a significantly higher risk of fractures [17,18]. Also, men with idiopathic hypogonadotropic hypogonadism and those with genetically-determined androgen resistance have below normal bone mass [19]. 

In our investigations, the level of OC did not depend on any hormonal parameters. OC is synthesized by osteoblasts, odontoblasts, and hypertrophic chondrocytes. Nonetheless, its role in the bone matrix remains unclear [20]. It is a highly specific marker of osteoblast activity, and thus bone turnover. Studies on animal models have shown that it regulates T synthesis in Leydig cells [21]. However, the available literature provides inconsistent data. The meta-analysis conducted by Zhong-Yu Liu et al. [22] demonstrated no significant difference in the levels of OC and T levels between men with primary osteoporosis and men without this disease when matched in terms of age. A clinical study of older men with sex hormone deficiency was described by Howard et al. [23]. They found that men with T or E2 deficiency were more likely to have lower bone mass. T deficiency in the study group resulted in faster bone mass loss, especially in the iliac bone. Zhong et al. [24], on the other hand, found that the level of OC was positively correlated with the level of T in men with hyperthyroidism. There are also reports on a positive correlation between the levels of OC and T and between the levels of T and CtX-I both in the general population and patients with bone disorders [25]. 

Our study also revealed the relationship between E2 and PINP levels in patients with TDS and without TDS. A similar observation in aging men was made by LeBlanc et al. [26]. Procollagen I amino terminal propeptide is a protein metabolized in the liver and released during collagen synthesis from type I procollagen. Serum PINP levels directly reflect the process and dynamics of collagen synthesis in osteoblasts [27]. It is worth emphasizing that PINP is regarded as a marker of early osteoblasts. When these cells are already mature and fully differentiated, a better marker is OC [28]. Nevertheless, the available literature provides few reports concerning the relationship between PINP and the hormonal parameters analyzed in our study. Our results showed that PINP levels depend on the levels of TT and E2. According to Xiao [29], on the other hand, serum PTH and SHBG levels are associated with biochemical markers of bone turnover: CtX-I, OC, and PINP. We also found that patients without TDS and with higher PINP levels had greater bone mass than their counterparts with lower PINP levels. This result should be treated with caution and no clinical conclusions should be drawn from it. However, it can be an indicator of where we should seek relationships concerning these issues. In a study of young men conducted by Välimäki et al. [30], they provided evidence that PINP levels in men can be dependent on the level of E2, which corresponds with our results obtained for older men. These authors also confirmed the relationship between the marker described here and the level of SHGB, which was not observed in our investigation. An important association was described by Falahati-Nini et al. [3], who carried out intervention research in which they inhibited the production of endogenous E2 and T using pharmacological agents and regulated the levels of these hormones by means of exogenous substitution. They found that taking E2 or T by men could prevent a decline in their OC levels, whereas only the administration of E2 could prevent a decrease in PINP levels. Thus, it can be concluded that E2 is the main regulator of the *new bone cell* formation―a marker of this process is PINP [31].

It is still under discussion which of the parameters―T and its derivatives or E2―have greater impact on bone turnover. In some publications the role of estrogens in the pathogenesis of male osteoporosis is emphasized [1,32]. It seems that, in the case of men, normal E2 levels are essential for achieving normal bone mass at a young age, but estrogens also play a vital role in bone metabolism in elderly men. In their study of 5995 men aged over 65 years, LeBlanc et al. [26] noticed that higher levels of bioavailable E2 entailed a greater risk of bone fractures (except for spinal vertebrae). They also demonstrated that low levels of bioT and high levels of SHBG did not increase the risk of fracture in men. Furthermore, they provided evidence that men with low levels of bioT and high levels of SHBG had an elevated risk of fractures, whereas men with low levels of bioE2 and bioT and high levels of SHBG were at the greatest risk.

In our study, the link between the levels of PTH and the levels of hormones and other parameters analyzed was not demonstrated. Nonetheless, there are publications showing that the selective lowering of T and E2 levels in men causes an increase in skeletal sensitivity to bone resorption by PTH [33].

It is worth emphasizing that TT deficiency is found in every fifth man with a vertebral fracture and in every third man with a femoral neck fracture. T deficiency in men is accompanied by a gradual process of trabecular bone loss. The average rate of BMD decrease in various locations in men is significantly lower than in women, and ranges from 0.5 to 1% annually [34]. Therefore, it seems important to seek new markers correlated with androgen levels.

It should be underlined that hormone levels are not the only factor that determine bone tissue metabolism. The role of other factors, such as obesity, inflammation, and immunological factors, should also be established. As the literature shows, a high fat mass might be a risk factor for osteoporosis and fragility fractures. Adipose tissue is the place of secretion of adipokines that influence the metabolic, skeletal, and cardiovascular systems [35]. Bone cells have several specific hormone receptors and can be regarded as a hormone target organ [36,37]. At the same time, OC has been proved to be a factor that may have an effect on body mass control and glucose metabolism [38]. Hence, the role of bone tissue in the regulation of a potential feedback loop between the skeleton and other endocrine organs should be taken into account [39]. It is also worth emphasizing that adiponectin inhibits *interleukin 6* (IL–6) and *tumor necrosis factor α* (TNF–α) production and thus reduces their proinflammatory effect [40]. The levels of adiponectin in obese patients are lower, which is why it has no anti-inflammatory effect. It is also an important element of the etiology of other conditions, such as asthma and cardiovascular disease [41,42].

Our study was limited in several ways. The main limitation was that it only involved men between 60 and 75 years of age. To get a comprehensive view of the relationship analyzed in our study, we should have also included older patients. Another limitation is the fact that E2 levels were determined using the ELISA method, possibly leading to inaccurate measurement. It would be better to determine this hormone by mass spectrometry. In the patients, we should have also determined 25(OH) Vitamin D levels since it is one of the main factors contributing to bone metabolism. The research should have also involved populations with normal and low levels of 25(OH)D. Finally, the ROC curve in our study was characterized by AUC = 0.656; this curve had no diagnostic, but only functional value. 

## 5. Conclusions

Analysis of sex hormones and biochemical bone markers, reflecting the quality of bone tissue in men, may suggest a relationship between these parameters. The role of similar future studies of patients diagnosed with osteoporosis should be underlined. More extensive research could provide results with clinical implications.

## Figures and Tables

**Table 1:** Relationship between the biochemical and anthropometric parameters, the levels of selected hormones, and the levels of *bone turnover markers* in patients with TDS and without TDS.

Variable	Patients without TDS *n* = 112		Patients with TDS *n* = 63		*p*
	X ± SD	Me	X ± SD	Me	
**Anthropometric parameters**					
**Age [years]**	66.86 ± 4.50	67.00	67.77 ± 4.54	68.00	0.294 ^2^
**Weight [kg]**	88.02 ± 13.96	88.00	97.88 ± 14.23	97.00	0.001 *^,1^
**BMI [kg/m^2^]**	28.84 ± 4.14	29.05	31.28 ± 4.18	30.86	0.003 *^,1^
**Hormonal parameters**					
**TT [ng/mL]**	5.67 ± 1.93	5.11	2.82 ± 0.76	3.16	0.001 *^,2^
**FT [ng/mL]**	0.09 ± 0.04	0.08	0.07 ± 0.03	0.07	0.004 *^,2^
**bioT [ng/dl]**	2.14 ± 0.95	1.98	1.52 ± 0.70	1.47	0.001 *^,2^
**E2 [pg/mL]**	89.19 ± 41.45	80.80	68.96 ± 36.86	54.14	0.003 *^,2^
**SHBG [nmol/L]**	60.33 ± 42.87	54.86	32.04 ± 23.94	24.23	0.001 *^,2^
**DHEAs [µg/mL]**	0.81 ± 0.58	0.64	0.82 ± 0.81	0.59	0.382 ^2^
**Markers of bone turnover**					
**CtX-I [ng/mL]**	0.44 ± 0.23	0.40	0.43 ± 0.18	0.39	0.885 ^2^
**PTH [pg/mL]**	38.37 ± 24.32	31.87	32.23 ± 17.07	30.54	0.172 ^2^
**OC [ng/mL]**	6.12 ± 4.30	5.45	6.56 ± 3.76	5.69	0.389 ^2^
**PINP [ng/mL]**	995.18 ± 869.42	834.25	700.95 ± 922.93	558.65	0.004 *^,2^
**Bone mineral density**					
**BMD [g/cm^2^] spine**	1.46 ± 0.24	1.43	1.41 ± 0.20	1.37	0.582 ^1^
**BMD [g/cm^2^] hip**	1.15 ± 0.18	1.14	1.08 ± 0.16	1.06	0.215 ^1^
**BMD [g/cm^2^]**	1.27 ± 0.15	1.29	1.24 ± 0.15	1.22	0.393 ^1^

(TDS, testosterone deficiency syndrome; n, number; X, arithmetic mean; SD, standard deviation; *p*, statistical significance; Me, median; BMD, bone mineral density; *BCM*, body cell mass; TT, total testosterone; FT, free testosterone; SHBG, sex hormone binding globulin; E2, estradiol; DHEAS, dehydroepiandrosterone sulfate; bioT, bioavailable testosterone; CtX-I, Serum Cross Laps; PINP, human procollagen I N-terminal peptide; PTH, parathyroid hormone; OC, osteocalcin; BMD, bone mineral density; * statistically significant parameter; *^1^*—Student’s *t*-test; *^2^*—Mann–Whitney U test).

**Table 2:** Multivariate linear regression analysis of the levels of hormonal parameters and proteins and the parameters of bone turnover in the groups with and without TDS. The results were adjusted for age and BMI.

Variable		Patients without TDS, *n* = 112				Patients with TDS, *n* = 63			
		*p*	Beta	−95.00%	+95.00%	*p*	Beta	−95.00%	+95.00%
**PINP**	TT ng/mL	0.001 *	0.389	0.175	0.604	0.458	0.141	−0.243	0.526
	FT ng/mL	0.060	0.234	−0.010	0.477	0.585	−0.118	−0.560	0.322
	bioT ng/dl	0.068	0.227	−0.018	0.471	0.484	−0.150	−0.585	0.284
	E2 pg/mL	0.002 *	0.360	0.132	0.588	<0.001 *	0.726	0.469	0.984
	SHBG nmol/L	0.153	0.177	−0.067	0.422	0.179	0.280	−0.136	0.696
	DHEA µg/mL	0.864	0.021	−0.220	0.262	0.850	0.035	−0.349	0.421
**PTH**	TT ng/mL	0.996	0.001	−0.232	0.233	0.122	0.275	−0.078	0.360
	FT ng/mL	0.492	−0.086	−0.334	0.162	0.545	−0.125	−0.543	0.593
	BioT ng/dl	0.730	−0.043	−0.293	0.206	0.623	−0.101	−0.515	0.314
	E2 pg/mL	0.258	0.137	−0.103	0.378	0.090	0.284	−0.054	0.623
	SHBG nmol/L	0.167	0.171	−0.073	0.416	0.133	0.296	−0.096	0.687
	DHEA µg/mL	0.265	−0.135	−0.374	0.104	0.208	−0.224	−0.579	0.132
**CtX-I**	TT ng/mL	0.354	0.107	−0.121	0.334	0.133	−0.277	−0.643	0.089
	FT ng/mL	0.313	−0.124	−0.367	0.119	0.091	−0.347	−0.753	0.059
	BioT ng/dl	0.149	−0.177	−0.419	0.065	0.057	−0.385	−0.781	0.012
	E2 pg/mL	0.274	0.131	−0.106	0.368	0.548	0.108	−0.255	0.471
	SHBG nmol/L	0.066	0.223	−0.015	0.462	0.428	−0.163	−0.578	0.251
	DHEA µg/mL	0.447	−0.091	−0.327	0.146	0.270	−0.203	−0.572	0.166
**OC**	TT ng/mL	0.201	0.145	−0.079	0.370	0.377	−0.160	−0.524	0.204
	FT ng/mL	0.938	−0.010	−0.253	0.234	0.298	−0.211	−0.618	0.196
	BioT ng/dl	0.705	−0.047	−0.291	0.198	0.280	−0.216	−0.618	0.185
	E2 pg/mL	0.463	0.087	−0.149	0.324	0.419	−0.141	−0.492	0.210
	SHBG nmol/L	0.115	0.190	−0.047	0.427	0.236	−0.236	−0.633	0.162
	DHEA µg/mL	0.251	−0.135	−0.368	0.098	0.257	−0.203	−0.561	0.155

*CI, confidence interval*; *p*, statistical significance; TT, total testosterone; FT, free testosterone; SHBG, sex hormone binding globulin; E2, estradiol; DHEAS, dehydroepiandrosterone sulfate; bioT, bioavailable testosterone; CtX-I, Serum Cross Laps; PINP, Human procollagen IN-terminal peptide; PTH, parathyroid hormone; OC, osteocalcin; *, *statistically significant parameter*.

**Table 3:** Comparison of the groups of patients with and without TDS divided with regard to PINP levels >619.333.

Variable	Patients with TDS					Patients without TDS				
	PINP Levels <619.333		PINP Levels >619.333		*p*	PINP Levels <619.333		PINP Levels >619.333		*p*
	X ± SD	Me	X ± SD	Me		X ± SD	Me	X ± SD	Me	
**Hormonal parameters**										
**TT** **ng/mL**	2.78 ± 0.82	3.14	2.87 ± 0.66	3.21	0.71	5.11 ± 1.52	4.57	6.15 ± 2.12	5.61	0.006 *
**FT** **ng/mL**	0.07 ± 0.03	0.08	0.06 ± 0.03	0.06	0.29	0.09 ± 0.04	0.07	0.10 ± 0.04	0.09	0.216
**bioT** **ng/dl**	1.60 ± 0.75	1.66	1.31 ± 0.59	1.26	0.28	1.97 ± 0.84	1.73	2.30 ± 1.02	2.13	0.206
**E2** **pg/mL**	57.91 ± 28.70	45.05	98.66 ± 41.69	95.77	0.01 *	78.08 ± 42.82	73.56	99.33 ± 38.22	84.57	0.007 *
**SHBG** **nmol/L**	29.66 ± 24.74	16.91	39.28 ± 23.32	30.04	0.11	57.83 ± 40.89	42.77	62.75 ± 45.12	48.12	0.590
**DHEA** **µg/mL**	0.86 ± 0.83	0.61	0.79 ± 0.85	0.51	0.74	0.86 ± 0.60	0.63	0.78 ± 0.57	0.70	0.480
**Bone mineral density**										
**BMD** **[g/cm^2^] spine**	1.44 ± 0.25	1.45	1.37 ± 0.12	1.33	0.52	1.40 ± 0.29	1.31	1.49 ± 0.22	1.44	0.867
**BMD** **[g/cm^2^] hip**	1.09 ± 0.19	1.09	1.07 ± 0.15	1.07	0.93	1.05 ± 0.17	1.03	1.19 ± 0.17	1.16	0.235
**BMD [g/cm^2^]**	1.25 ± 0.19	1.24	1.23 ± 0.12	1.22	0.78	1.22 ± 0.16	1.26	1.29 ± 0.14	1.30	0.032 *

PINP, Human procollagen N-terminal peptide; X, arithmetic mean; SD, standard deviation; Me, median; p, statistical significance; TT, total testosterone; FT, free testosterone; SHBG, sex hormone binding globulin; E2, estradiol; DHEAS, dehydroepiandrosterone sulfate; bioT, bioavailable testosterone; BMD, *bone mineral density*; *, *statistically significant parameter*.
